# Effect of Flow Length on Pressure and Measurement of PEMFC Temperature by Using Thin-Film Thermocouples

**DOI:** 10.3390/mi16050535

**Published:** 2025-04-29

**Authors:** Huijin Guo, Zhihui Liu, Xingyu Li, Xingshu Wang, Maopeng Zhang, Shiqi Zhang, Zixi Wang, Wanyu Ding

**Affiliations:** 1 School of Automotive Studies, Tongji University, Shanghai 201804, China; 2 State Key Laboratory of Tribology in Advanced Equipment, Tsinghua University, Beijing 100084, China; 3 College of Materials Science and Engineering, Dalian Jiaotong University, Dalian 116028, China; 13830097473@163.com; 4 School of Science, Tianjin Chengjian University, Tianjin 300384, China; 18077772816@163.com; 5 School of Mechanical Engineering, Dalian Jiaotong University, Dalian 116028, China; dljtwxs@163.com (X.W.); sqzhang20011203@126.com (S.Z.)

**Keywords:** proton exchange membrane fuel cell, flow length, pressure distribution, thin-film thermocouple, operating temperature

## Abstract

Based on the COMSOL simulation software (v.6.1), this paper systematically investigates the influence law of runner length on the velocity and pressure distribution of cathode and anode gas runners in proton exchange membrane fuel cells (PEMFCs), and experimentally verifies the measurement effect of thin-film thermocouples on the operating temperature of PEMFCs. The simulation results show that the maximum pressure of the cathode and anode increases nonlinearly with the increase in the runner length, while the velocity distribution remains stable; the shortening of the runners significantly reduces the friction loss along the flow path and optimizes the matching of the permeability of the porous medium. In addition, the NiCr/NiSi thin-film thermocouple prepared by magnetron sputtering exhibits high accuracy (Seebeck coefficient of 41.56 μV/°C) in static calibration and successfully captures the dynamic response characteristics of temperature in PEMFC operation. This study provides a theoretical basis and experimental support for the optimization of fuel cell flow channel design and temperature monitoring technology.

## 1. Introduction

As an efficient and clean energy conversion device, a proton exchange membrane fuel cell (PEMFC) holds great potential for applications in new energy vehicles, distributed power generation, and portable power supplies [1,2]. Its core advantages lie in its low-temperature fast startup, high energy density, and zero-carbon emission characteristics, and it is regarded as one of the key technologies to achieve the goal of carbon neutrality. However, the commercialization of PEMFCs still faces many challenges, among which the issues of runner design and thermal management are the key bottlenecks for performance improvement and lifetime extension [3,4].

Recent studies have extensively explored the aforementioned issues through both theoretical analysis and practical investigation. Currently, numerical simulations—such as finite element modeling—and experimental validation remain the two most widely adopted and effective methodologies. These approaches have enabled researchers to gain deeper insights into the underlying mechanisms and to evaluate the performance of proposed designs under realistic conditions. Qin et al. [5]. conducted a novel simulation study on the dynamic behavior of liquid water in three-dimensional multichannels with various shape designs, and the results showed that the first channel in the multichannel drained slower than the second and third channels. Do et al. [6]. investigated the effects of obstacles and channel depth on the performance of a PEMFC in a serpentine flow channel by Computational Fluid Dynamics (CFDs), and the results showed that the effect of channel depth was greater than that of the presence of obstacles in the flow channel. Chen et al. [7] developed and systematically evaluated six runner configurations by CFD simulation, and they used parametric analysis combined with an orthogonal experimental design to investigate the effects of the number of subrunners (N), angle (α), width (d), and spacing (L). Brakni et al. [8] developed a 3D CFD model of a PEMFC with various flow field designs, and they considered the effect of different runner shapes on the operating state of the PEMFC. Simulation is likewise one way to understand the operating temperature of the PEMFC. Chen et al. [9] developed an integrated PEMFC system oriented control model with parameter identification, and their proposed PEMFC system contains a gas supply subsystem, a hydrogen supply subsystem, and a thermal management subsystem, which can reflect the temperature change during current perturbation. Wang et al. [10] investigated the effects of cooling rate, current density, and other issues on the operating temperature of PEMFCs. Arat et al. [11] investigated how the modeled PEM fuel cell is affected by transients at sub-zero (0, −5, −10, −11, −12, −13, −14, −15), and their detailed evaluation of transients of the PEMFC under extreme cold conditions revealed that parameters such as energy, heat flow, voltage, and wall temperature, with changes in energy, shift over time. However, simulations tend to optimize conditions that cause their results to differ from reality. The operating temperature of the PEMFC can be better measured experimentally compared to the flow channel [12]. Lee et al. [13] conducted distributed in situ temperature measurements on a polybenzimidazole-based high-temperature proton exchange membrane fuel cell. They embedded a total of 16 T-type thermocouples into the anode and cathode flow plates to achieve this. However, they disrupted the structure of the cell, which is an undesirable behavior, despite the fact that they illustrated that there would be no effect on the operation of the cell. Lee et al. [13] developed an in-line thermocouple with a diameter of 49 μm for in-plane temperature monitoring, which enabled multipoint measurements at the expense of longer assembly time. Meanwhile, Ali et al. [14] proposed a T-type (copper–calcium) thin-film thermocouple (TFTC) array on 75-μm-thick Kapton foil for measurement of the high-temperature conditions in a PEMFC, and Sugimoto et al. [15] applied an array of Au–Ni thin-film thermocouples on poly(p-xylene) film for internal temperature monitoring of a PEMFC. In summary, there are studies on flow paths that mostly consider shape, but there is no clear indication of the relationship between length and pressure. For temperature measurement, most of the studies in the previous years used filament thermocouples, which would destroy the structure of the PEMFC. In recent years most of them use thin-film thermocouples for temperature measurement, but their cost is high.

Based on the above background, this paper adopts a combination of multi-physics field simulation and experimental validation to systematically study the influence law of the runner length on the cathode gas flow velocity and pressure distribution of PEMFCs. At the same time, NiCr/NiSi thin-film thermocouples are prepared by magnetron sputtering technology, and their temperature measurement reliability is verified under the complex working conditions of PEMFCs by combining static calibration and dynamic temperature measurement experiments. The research results can provide theoretical support and technical reference for the optimized design of fuel cell runner structure and intelligent upgrading of thermal management strategy.

## 2. Influence of Runner Length on the Velocity as Well as the Pressure of the Bipolar Gas Flow Channel

### 2.1. Model Definition

**Figure 1:**
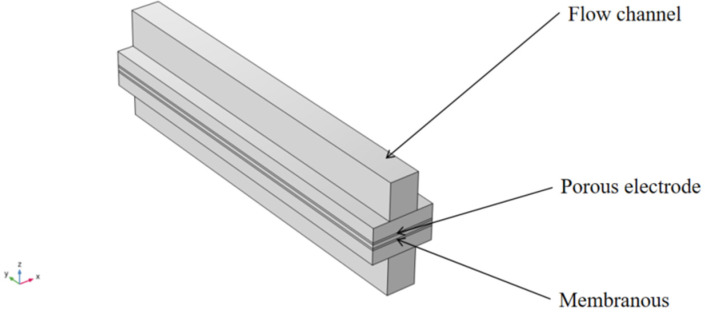
Geometric modeling.

The geometric mode model is shown in Figure 1.

### 2.2. Principles

At the anode, the hydroxide reaction is as described in [16].

(1) $$H_{2}\Rightarrow 2H^{+}+2e^{-}$$

The local current density expression used for the hydroxide reaction is as follows:

(2) $$i_{a}=i_{0,ref,a}\left(\frac{P_{H_{2}}}{P_{ref}}\exp\left(\frac{\alpha_{a,a}}{RT}F\eta_{a}\right)-\exp\left(-\frac{\alpha_{c,a}}{RT}F\eta_{a}\right)\right)$$

where $P_{H_{2}}$ is the local partial pressure of oxygen and $P_{ref}$ is a reference pressure of 1 atm. *a* is the overpotential of the reaction gas at atmospheric pressure relative to the reaction equilibrium potential.

At the cathode, oxygen reacts with protons to form water with the following reaction equation.

(3) $$O_{2}+4H^{+}+4e^{-}\Rightarrow 2H_{2}O$$

The current density expression is used for the oxygen reduction reaction, as follows:

(4) $$i_{c}=i_{0,ref,c}\left({\left(\frac{P_{H_{2}O}}{P_{ref}}\right)}^{2}\exp\left(\frac{\alpha_{a,c}}{RT}F\eta_{c}\right)-\frac{P_{O_{2}}}{P_{ref}}\exp\left(-\frac{\alpha_{c,c}}{RT}F\eta_{c}\right)\right)$$

where $P_{O_{2}}$ and $P_{H_{2}O}$ are the local partial pressures of oxygen and water vapor, respectively.

### 2.3. Boundary Conditions

The hydrogen fuel cell model is constructed through the “Hydrogen Fuel Cell” interface, two “Free and Porous Media Flow” interfaces, and the “Reaction Flow, Hydrogen Phase” and “Reaction Flow, Oxygen Phase” multiphysics field nodes. The boundaries of the anode gas diffusion layer towards the flow channel are set to zero potential, while the corresponding boundaries on the cathode side are set to cell potential. All other external boundaries are insulated.

In the “Hydrogen Fuel Cell” interface, the Maxwell–Stefan equations are utilized to calculate the mass fractions of substances within the flow channel, gas diffusion layer, and gas diffusion electrode. It is important to note that the anode side consists of hydrogen and water, whereas the cathode side contains oxygen, water, and nitrogen. Mole fractions are defined at the channel inlet, while exit conditions are applied at the channel outlet. For all other external boundaries, zero flux conditions are employed.

The velocity (u) and pressure (p) are modeled using two interfaces for “free and porous media flow.” In the flow channel, the Navier–Stokes equations are applied, whereas the Brinkman equations are used for the gas diffusion layer and gas diffusion electrode. Density, viscosity, velocity, and pressure, as well as net mass sources and sinks, are coupled to the “Hydrogen Fuel Cell” interface through the “Reactive Flow, Hydrogen Phase” and “Reactive Flow, Oxygen Phase” multiphysics field nodes.

At the inlet boundary of the flow channel, a laminar inflow velocity distribution is specified. At the outlet boundary, a pressure boundary condition is applied. To simulate multiple parallel channels, symmetric boundary conditions are imposed along the long edges of the gas diffusion layer and gas diffusion electrode. No-slip boundary conditions are applied to all other wall boundaries.

### 2.4. Results

**Figure 2:**
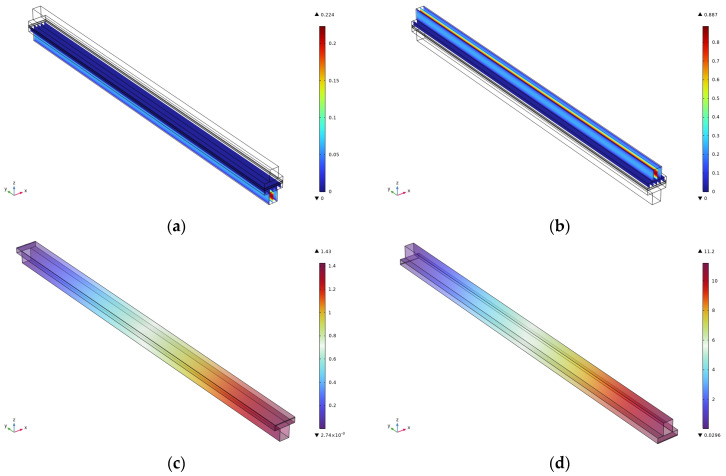
Simulation results for a runner length of 3 cm. (**a**) Cathode speed; (**b**) Anode speed; (**c**) Cathodic pressure; (**d**) Anode pressure.

This paper analyzes the effect on the velocity and pressure by comparing the flow channel lengths of 1 cm, 1.5 cm, 2 cm, 2.5 cm, and 3 cm. The velocity, as well as pressure cloud diagrams, for the cathode and anode at the flow channel of 3 cm are shown in Figure 2. The results show that for the anode velocity field (Figure 2a), the reacting gas presents a uniform flow distribution characteristic in the flow channel, and this flow characteristic is conducive to enhancing the mass transfer efficiency at the gas–liquid–solid three-phase interface. The coupled analysis of PIV flow field visualization and CFD simulation reveals that there is no obvious flow separation or vortex structure in the flow channel, and this laminar-dominated flow pattern can effectively inhibit the formation of a local concentration gradient, thus reducing the effect of concentration polarization on the electrochemical performance. The cathode-side velocity field (Figure 2b shows a significant velocity gradient in the inlet section, and this phenomenon is closely related to the high Reynolds number flow characteristics and the abrupt change of the flow channel geometry parameters. The boundary layer theory analysis reveals that the accelerated flow in the inlet section leads to an enhanced velocity slip effect on the surface of the gas diffusion layer, which may affect the mass transfer process of oxygen to the catalyst layer. It is noteworthy that the velocity field tends to stabilize after the 60% position of the runner length, when the weight of the influence of the Schmidt number on the substance transport increases significantly. The numerical simulation results of the anode pressure field, Figure 2c, show that there is a pressure drop in the axial direction of the flow channel, with the maximum pressure reaching 1.43 Pa. This low-pressure gradient characteristic is mainly attributed to the optimized design of the flow channel cross-section and the precise control of the surface roughness, which ensures the spatial uniformity of the transverse mass-transfer fluxes within the gas diffusion layer. The cathode pressure field (Figure 2d) is characterized by an obvious pressure gradient in the inlet section, with the maximum pressure reaching 11.2 Pa. This gradient distribution shows a strong correlation with the spatial variation of the permeability of the porous medium. The modified model analysis of the Darcy–Forchheimer equation reveals that the insufficient pressure matching at the interface of the flow channel–gas diffusion layer is the key factor leading to the increase of the local mass transfer resistance.

**Figure 3:**
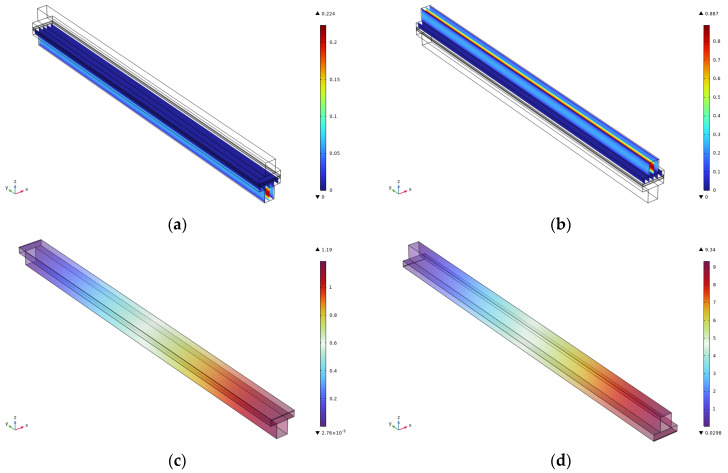
Simulation results for a runner length of 2.5 cm. (**a**) Cathode speed; (**b**) Anode speed; (**c**) Cathodic pressure; (**d**) Anode pressure.

The velocity, as well as pressure cloud, of the cathode and anode at the flow channel of 2.5 cm is shown in Figure 3. It can be seen that the cathode and anode velocities at 2.5 cm are consistent with those at 3 cm, but the maximum pressure of the cathode decreases from 1.43 Pa at 3 cm to 1.19 Pa at 2.5 cm, and the maximum pressure of the anode decreases from 11.2 Pa at 3 cm to 9.34 Pa at 2.5 cm, which is the main reason for the evolution of the pressure characteristics. The change of flow energy dissipation mechanism is triggered by the change of flow geometry parameters: According to the energy conservation principle of Bernoulli’s equation, the reduction of runner length leads to the linear reduction of friction loss along the course, in which the friction factor is a function of the Reynolds number so that it is easier to maintain the laminar flow state in the short runner. The synergistic adjustment of the cross-sectional area of the runner and the length alters the local acceleration effect, and the increase of flow velocity due to the shrinking of the runner will enhance the dynamic pressure component and promote the static pressure component when the mass flow rate is constant. When the mass flow rate is constant, the velocity increase caused by the flow channel constriction will enhance the dynamic pressure component, and the static pressure component will be reduced accordingly to maintain the conservation of the total pressure The relatively large pressure drop on the cathode side is related to the non-uniform distribution of permeability in the gas diffusion layer, and the shortening of the flow channel attenuates the coupling effect of Darcy flow and Fuchsheimer flow in the porous medium, which in turn decreases the gradient of the permeation resistance.

**Figure 4:**
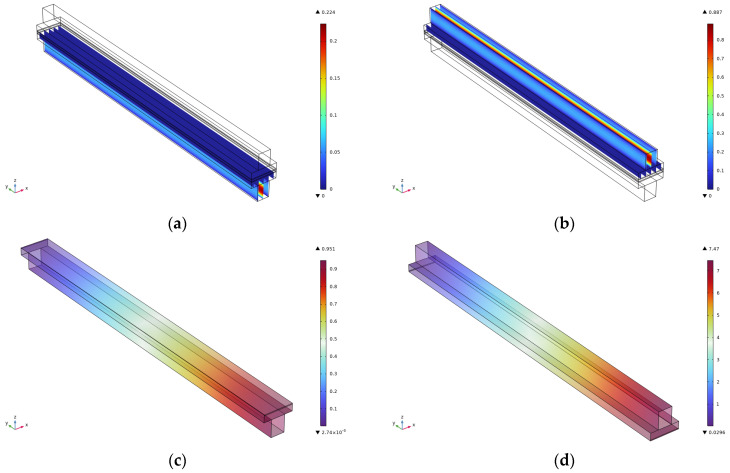
Simulation results for a runner length of 2 cm. (**a**) Cathode speed; (**b**) Anode speed; (**c**) Cathodic pressure; (**d**) Anode pressure.

The velocity, as well as pressure cloud, of the cathode and anode at the flow channel of 2 cm is shown in Figure 4. From the figure, it can be seen that the velocity of the cathode and anode at the 2 cm runner is consistent with that at the 3 cm runner and 2.5 cm runner, but the maximum pressure of the cathode decreases from 1.43 Pa at the 3 cm runner to 0.951 Pa at the 2 cm runner, and the maximum pressure of the anode decreases from 11.2 Pa at the 3 cm runner to 7.47 Pa at the 2 cm runner.

**Figure 5:**
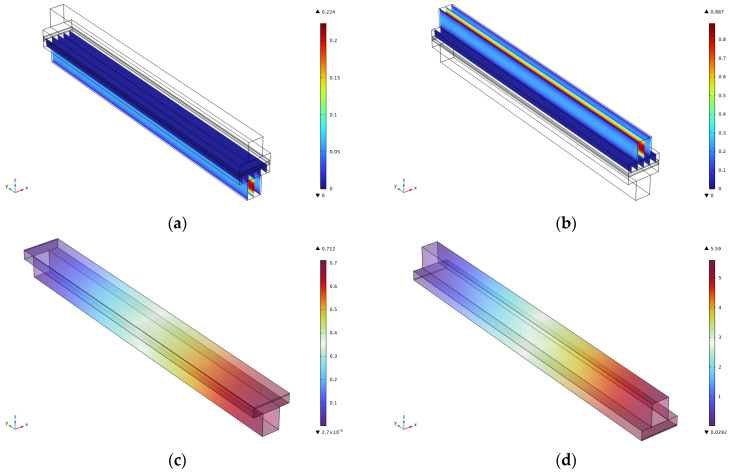
Simulation results for a runner length of 1.5 cm. (**a**) Cathode speed; (**b**) Anode speed; (**c**) Cathodic pressure; (**d**) Anode pressure.

The velocity, as well as pressure cloud, of the cathode and anode at the 1.5 cm runner is shown in Figure 5. From the figure, it can be seen that the cathode and anode velocities at the 1.5 cm runner are consistent with those at the 3 cm runner, 2.5 cm runner, and 2 cm runner, but the maximum cathode pressure decreases from 1.43 Pa at the 3 cm runner to 0.712 Pa at the 1.5 cm runner, and the maximum anode pressure decreases from 11.2 Pa at the 3 cm runner to 5.59 Pa at the 1.5 cm runner.

**Figure 6:**
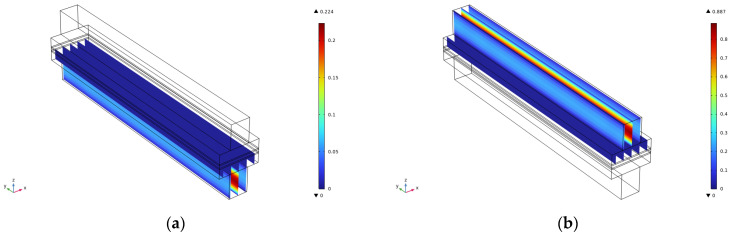

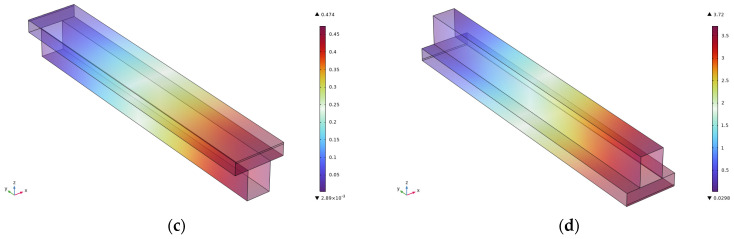
Simulation results for a runner length of 1 cm. (**a**) Cathode speed; (**b**) Anode speed; (**c**) Cathodic pressure; (**d**) Anode pressure.

The velocity, as well as pressure cloud, of the cathode and anode at the 1 cm runner is shown in Figure 6. From the figure, it can be seen that the cathode and anode velocities at the 1 cm runner are consistent with the other four runner lengths, but the maximum cathode pressure decreases from 1.43 Pa at the 3 cm runner to 0.474 Pa at the 1 cm runner, and the maximum anode pressure decreases from 11.2 Pa at the 3 cm runner to 3.72 Pa at the 1 cm runner.

**Figure 7:**
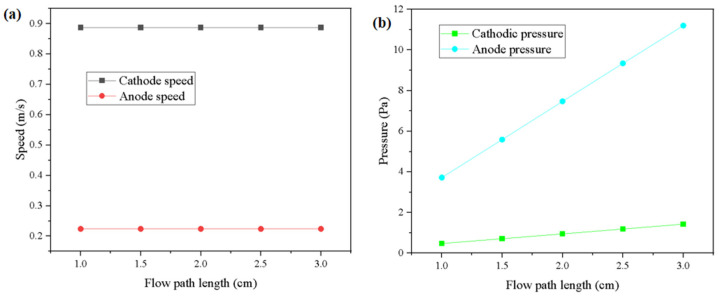
Velocity vs. pressure results for the different runner lengths. (**a**) Speed change; (**b**) Pressure change.

According to the simulation results, it can be concluded that as the flow channel length increases, the pressure within both the anode and cathode channels gradually increases in a nonlinear manner. As shown in Figure 7a, the velocity at both the anode and cathode remains essentially constant regardless of runner length. This indicates that the mass flow rate is conserved and not significantly impacted by the geometric change, confirming that the flow regime remains in a laminar and fully developed state across the studied lengths.

However, Figure 7b shows a clear and systematic increase in maximum pressure as the runner length increases. Specifically, the cathode maximum pressure increases by 50.21% when the length increases from 1 cm to 1.5 cm, 33.57% from 1.5 cm to 2 cm, 25.13% from 2 cm to 2.5 cm, and 20.17% from 2.5 cm to 3 cm. Similarly, the anode pressure shows increases of 50.27%, 33.63%, 25.03%, and 19.91% for the same intervals. This decelerating growth trend suggests that the relationship between pressure and runner length follows a diminishing return pattern, which is characteristic of friction-dominated flow in confined channels. As the gas flows along the channel, it experiences continuous shear stress along the channel walls and within the porous gas diffusion layer (GDL). The increase in runner length directly extends the friction path, thereby causing more energy loss in the form of a pressure drop. The rate of increase slows down because, in longer channels, the flow becomes more stabilized and the incremental friction per unit length contributes less to the overall pressure rise.

In addition to the channel walls, gases interact with the porous GDL and catalyst layers. These regions exhibit distributed flow resistance, especially when their permeability is non-uniform. As the flow progresses along a longer runner, the cumulative impedance from these porous regions becomes significant, leading to a gradual buildup of static pressure, particularly at the anode. The gas flow undergoes acceleration and deceleration due to minor geometric irregularities and transitions between the free-flow and porous regions. Although overall the flow is laminar, the inertial effects near the inlet cause localized increases in velocity gradients, contributing to sharper pressure gradients in short runners. In contrast, longer runners allow these effects to dissipate over distance, redistributing the pressure more gradually. Despite the cathodic gas flow rate being higher than that of the anode, the simulation consistently shows higher pressure at the anode. This apparent contradiction arises from the nature of the electrochemical reactions and system design. The hydrogen oxidation reaction at the anode is fast and requires minimal reactant concentration, but high hydrogen supply pressure is essential to overcome the diffusion resistance in the GDL and ensure uniform delivery to the catalyst layer. This structural demand naturally leads. Conversely, the cathode reaction involves oxygen reduction, which is kinetically slower and more sensitive to local concentration drops. To ensure sufficient supply, the system increases the oxygen flow rate rather than pressure. Additionally, the cathode side often experiences higher water production and liquid saturation, which may further increase local mass transfer resistance, but this resistance is often compensated by flow velocity rather than static pressure. The interplay between the runner length, pressure gradient, and mass transfer efficiency implies that an optimal runner length must strike a balance: long enough to provide sufficient residence time for gas exchange, but short enough to minimize pressure losses and associated parasitic power.

## 3. Thin-Film Thermocouples for Measuring PEMFC Operating Temperatures

This section investigates the measurement of the operating temperature of a proton exchange membrane fuel cell (PEMFC) using a prepared NiCr/NiSi thin-film thermocouple. Since the flow channel of the PEMFC needs to maintain a continuous flow of gas, the placement of the temperature sensor is critical. Improper sensor placement may interfere with the normal operation of the fuel cell, so how to accurately measure the temperature without affecting the gas flow is a key challenge in the thermal field measurement of the PEMFC.

### 3.1. Principle of the Operation of Thin-Film Thermocouples

Thin-film thermocouples typically consist of two different conductive materials deposited onto a substrate by thin-film deposition techniques and form a hot end and a cold end. This structure allows the thermocouple to fit closely to the target surface for highly accurate temperature measurement. When the temperature of the hot end is higher than that of the cold end, a thermoelectric potential is generated due to the different electron diffusion capacities of the different materials

**Figure 8:**
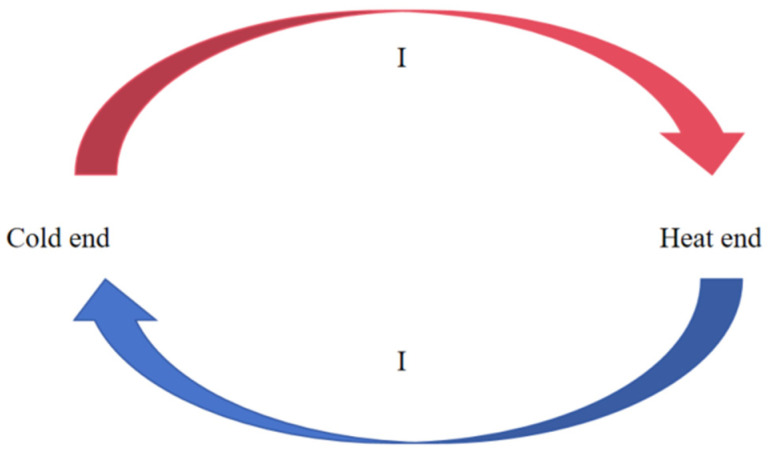
Principle of the operation of thin-film thermocouples.

(5) E=S⋅ΔT

where *S* is the Seebeck coefficient of the material and Δ*T* is the temperature difference between the hot and cold end. This voltage signal can be amplified by a highly sensitive measuring system and is thus used for temperature calculations. The specific working process is shown in Figure 8. Thin-film thermocouples have the advantages of fast response, high spatial resolution, and that they can be integrated. Since the thickness of the film is generally in the nanometer to micrometer level, the heat capacity is small, and it can respond quickly to temperature changes, which is suitable for temperature measurement in tiny areas.

### 3.2. Thin-Film Thermocouple Structure Design

**Figure 9:**
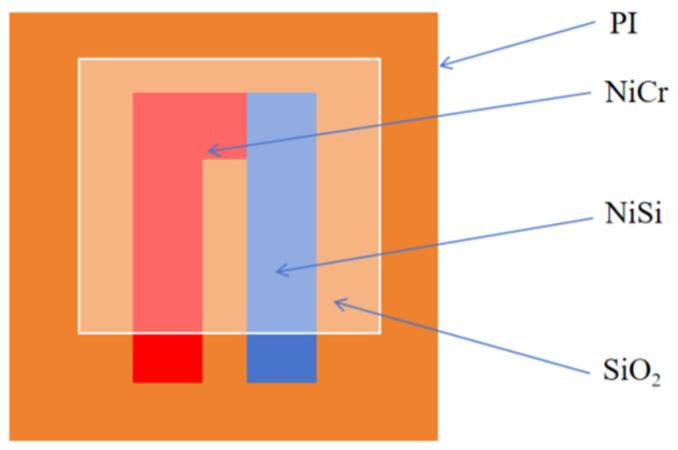
Thin-film thermocouple structure.

As mentioned earlier, improper placement of the temperature sensor may interfere with the normal operation of the PEMFC. Compared with traditional thermocouple wires, thin-film thermocouples have the advantages of small size and high measurement accuracy, which makes them more suitable for monitoring the internal temperature of fuel cells. However, the presence of large amounts of oxygen and hydrogen during the operation of a PEMFC may affect the performance of the sensor, so it is necessary to protect the thin-film thermocouple with appropriate coating to ensure its long-term stable operation in harsh environments. Considering the above factors, polyimide (PI) is selected as the substrate of the thin-film thermocouple in this paper, and NiCr/NiSi films are sequentially deposited on its surface to construct a high-precision temperature sensor. In addition, in order to improve the stability of the thin-film thermocouple in the fuel cell environment and prevent the corrosion caused by oxygen, SiO_2_ is used as a protective layer. The specific structure design is shown in Figure 9.

### 3.3. Thin-Film Thermocouple Process Selection

Thin films are prepared by a variety of processes, including screen printing [17,18], photolithography [19,20], chemical vapor deposition [21,22], and so on. Different processes have their own advantages and disadvantages. For example, screen printing is a simple and low-cost process, but its pattern accuracy is poor, which is suitable for thick films but not for high-precision micro- and nano-structures; photolithography can realize high-precision patterns, but it is a complicated process with cumbersome steps, and it is costly for large-area preparation; and CVD is capable of preparing high-quality homogeneous thin films, but it usually requires high-temperature environments, and the choice of materials is limited.

In contrast, magnetron sputtering [23,24] is an excellent thin-film deposition method, which can be deposited at low temperatures and is suitable for temperature-sensitive substrate materials. Magnetron sputtered films have high uniformity and precise thickness control, and can be used for large-area deposition. In addition, the process is capable of preparing films with high densities and high adhesion, which improves the mechanical strength and stability of the films. Finally, magnetron sputtering can be applied to a wide range of materials and is able to optimize the film layer properties by adjusting the process parameters, giving it a significant advantage in microelectronics, sensors, and high temperature environment applications. Therefore, in this paper, magnetron sputtering was selected for the preparation of the thin-film thermocouples, and the specific process parameters are shown in Table 1 [25].

### 3.4. Thin-Film Thermocouple Calibration

Prepared thin-film thermocouples require static calibration to determine their temperature–voltage conversion relationship to ensure that they accurately reflect temperature changes in practical applications. During the calibration process, the output voltage of the thin-film thermocouple is compared to a known temperature standard source to establish an accurate temperature–voltage characteristic curve. This process involves gradually heating or cooling the thin-film thermocouple and recording the voltage values at different temperatures to obtain a voltage–temperature relationship model through data processing, which is ultimately used for temperature measurement and conversion in real-world applications.

**Figure 10:**
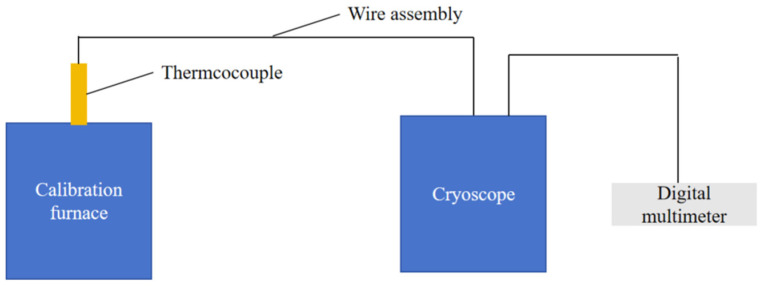
Static calibration schematic.

The static calibration system is depicted in Figure 10. A bipolar plate sample, equipped with a thin-film thermocouple, is positioned inside the calibration furnace. The furnace opening is insulated with asbestos material to minimize heat loss. The thermocouple leads extend outside the furnace, with one end connected to a freezing point instrument set at 0 °C, and the other end linked to a digital multimeter via a copper wire. The digital multimeter records the electromotive force (emf) readings, which are then used to calculate the Seebeck coefficient. In this study, the temperature range was set between 50 and 200 °C, and the calibration process involved seven evenly spaced temperature holding points, each maintained for five minutes [26].

**Figure 11:**
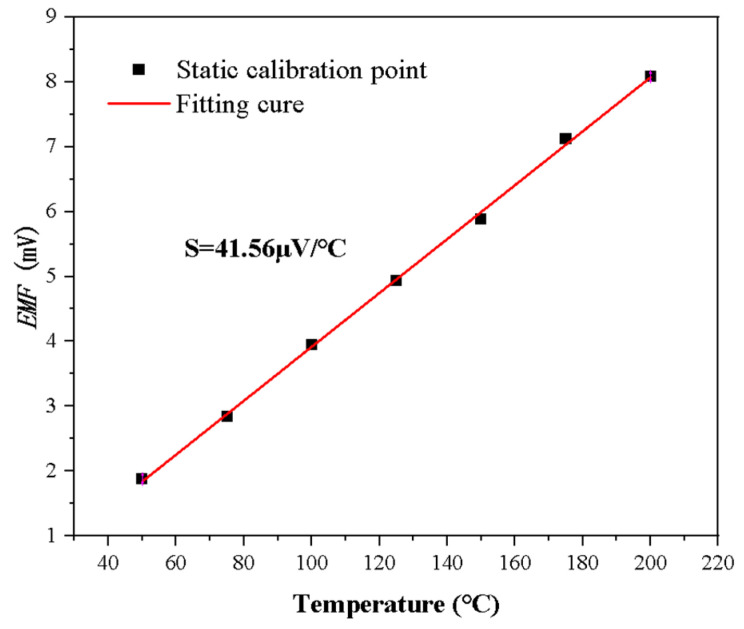
Thin-film thermocouple static calibration results.

The static calibration results of the NiCr/NiSi thin-film thermocouple are shown in Figure 11. In this study, the temperature of the thin-film thermocouples was controlled using a high-precision calibration oven with the temperature range set from 50 °C to 200 °C. To ensure accuracy and consistency of the measurements, seven uniformly distributed temperature points were selected, each of which was kept stable at the set temperature for five minutes. Each of these temperature points covered the temperature range studied, ensuring that the temperature response characteristics of the thin-film thermocouples could be fully evaluated.

At the specified temperature points, the thermoelectric potential output of the NiCr/NiSi thin-film thermocouple was precisely measured and documented. The Seebeck coefficient (S) for this thin-film thermocouple was determined through linear regression of the experimental data. This coefficient indicates the thermoelectric characteristics of the thin-film thermocouple, specifically the relationship between the electromotive force generated by the material and the temperature difference caused by a temperature gradient. Additionally, the homemade NiCr/NiSi thin-film thermocouple was statically calibrated using the same experimental procedure, and its Seebeck coefficient was obtained. Figure 11 presents the static calibration results for the thin-film thermocouples, clearly demonstrating the changes in electric potential and the corresponding Seebeck coefficient. For the NiCr/NiSi thin-film thermocouple and the NiSi thin-film thermocouple, the Seebeck coefficient was found to be S = 41.56 μV/°C across different temperatures.

### 3.5. PEMFC Operating Temperature Measurements

**Figure 12:**
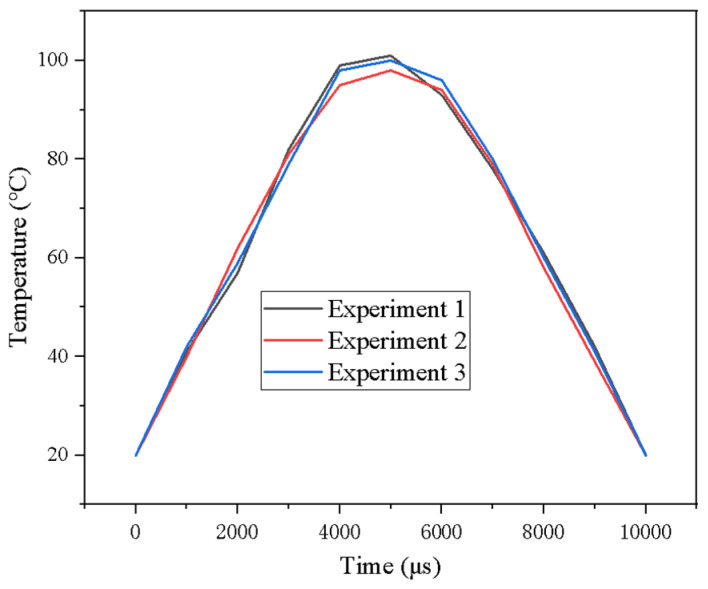
PEMFC operating temperature measurement results.

The actual operating temperature data of the PEMFC measured using a thin-film thermocouple is shown in Figure 12. For the convenience of observing the trend, only the temperature data of 0 μs, 1000 μs, …, 10,000 μs were recorded, and measurement was repeated three times. As can be seen in Figure 12, the temperature variation with time shows a typical thermal response curve. At the initial stage (0–5000 μs), the temperature increases gradually, starts to decrease after reaching the peak (about 5000 μs), and returns to the initial temperature (20 °C) at 10,000 μs. This trend indicates that the proton exchange membrane fuel cell (PEMFC) went through distinct heating and cooling phases during startup and operation. The data from the three experiments showed a high degree of consistency at most time points, and especially good repeatability during the temperature rise and fall phases. This indicates that the experimental conditions and measurement methods are highly controllable and stable, thus ensuring the reliability of the data. Rising phase: the temperature rises rapidly from 20 °C to about 100 °C, indicating that there is a significant exothermic effect of the chemical reaction during the start-up phase of the PEMFC. Peak phase: the temperature peaks at about 5000 μs (~100 °C), a phenomenon that may reflect that the chemical reaction rate has reached its maximum or that the system is in thermal equilibrium. Decrease phase: the temperature then gradually decreases, suggesting that the intensity of the chemical reaction is weakening or that the system is beginning to cool down. Near the peak (~5000 μs), there is a slight fluctuation in temperature. This fluctuation may be due to factors such as transient fluctuations in the chemical reaction rate; inhomogeneity of heat transfer within the system; measurement errors; or external disturbances.

## 4. Conclusions

In this study, a comprehensive simulation and experimental investigation was conducted to elucidate the influence of flow channel length on the internal flow characteristics and thermal behavior of proton exchange membrane fuel cells (PEMFCs). The major contributions and findings of this work are summarized as follows:

1. A multiphysics simulation framework was used to reveal the non-linear relationship between flow channel length and internal pressure distribution. The maximum anode pressure increased from 3.72 Pa (at 1 cm) to 11.2 Pa (at 3 cm), while the flow velocity remained nearly constant across all channel lengths, exhibiting stable laminar flow characteristics. It was further demonstrated that shorter channels help suppress concentration polarization and enhance gas–liquid–solid interface mass transfer by minimizing frictional losses and improving permeability coupling within the porous layers.
2. A NiCr/NiSi thin-film thermocouple was successfully fabricated using magnetron sputtering. Static calibration yielded a high Seebeck coefficient of 41.56 μV/°C with excellent linearity and a calibration error below 1.5%. The incorporation of a SiO₂ protective layer significantly improved its stability under the PEMFC’s humid and oxidizing environment. This sensor offers a reliable, minimally intrusive solution for temperature monitoring in high-performance electrochemical systems.
3. Dynamic measurements of the PEMFC operating temperature revealed a characteristic thermal response curve—rapid heating to ~100 °C followed by gradual cooling—that was consistent across three repeated experiments, with fluctuation within 5%. These results confirm the sensor’s robustness and suitability for real-time thermal diagnostics in PEMFCs.

The outcomes of this study provide actionable insights for both academic researchers and industrial practitioners. For PEMFC system designers, the established relationship between runner length and pressure distribution offers a practical design guideline to optimize flow resistance and reaction efficiency, supporting the development of more compact and high-performance fuel cell stacks. For sensor developers, the validated integration method of NiCr/NiSi thin-film thermocouples enables real-time, high-resolution temperature monitoring without disrupting the internal structure of the fuel cell. Additionally, for researchers focusing on thermal management, the calibrated response curves and dynamic temperature data serve as valuable reference benchmarks for enhancing model accuracy and optimizing control strategies.

In summary, by combining flow simulation, sensor design, and temperature validation, this study offers a complete framework that bridges theoretical modeling and practical engineering. The findings contribute new knowledge to the optimization of PEMFC flow channels and thermal sensing, and are expected to support further research into intelligent fuel cell management, compact system integration, and next-generation microsensors for harsh environments.

## Figures and Tables

**Table 1:** Thin-film thermocouple process parameters.

Experimental Parameters	NiCr Membrane	NiSi Membrane	SiO_2_ Membrane
Target material	NiCr target	NiSi target	SiO_2_ target
Target-base distance	120 mm	120 m	120 mm
Working gas	Ar	Ar	Ar/O_2_
Working pressure	0.7 Pa	0.7 Pa	0.6 Pa
Flow	20 sccm	20 sccm	20/5 sccm
Inversion time	1 μs	1 μs	1 μs
Pulse frequency	100 kHz	100 kHz	100 kHz
Sputtering power density	1.9 W cm^−2^	1.9 W cm^−2^	0.83–5.00 Wcm^−2^
Deposition time	35 min	41 min	3.5 h

## Data Availability

The original contributions presented in this study are included in the article. Further inquiries can be directed to the corresponding authors.
